# Efficiency and equity of water transfers to megacities: reconfiguring urban water security in Istanbul

**DOI:** 10.7717/peerj.21241

**Published:** 2026-06-17

**Authors:** Frances Varty Bayar, İrem Daloğlu Çetinkaya

**Affiliations:** 1 Institute of Environmental Sciences, Bogazici University, Istabul, Turkey

**Keywords:** Water security, Interbasin transfer, Socio-hydrology, Urban water governance

## Abstract

**Background:**

Rapid urbanization, population growth, and climate-induced water stress have made interbasin water transfers (IBTs) a central strategy for augmenting urban water supplies worldwide. Istanbul, Türkiye’s largest metropolitan area, relies heavily on water transfers to meet its rising demand. However, concerns around the long-term sustainability, efficiency, and equity of these transfers, particularly their impact on donor regions, remain underexamined. This study investigates the hydrological and social performance of Istanbul’s major water transfers, with a focus on balancing urban water security with environmental and regional justice considerations.

**Methods:**

A socio-hydrological case study approach was used to assess three major water transfers supplying Istanbul from Düzce, Tekirdağ, and Kırklareli provinces between 2000 and 2023. Two quantitative indicators were applied: the Natural Efficiency Index, which compares transfer volumes to renewable freshwater availability in both donor and recipient basins; and the Stress Relief Index, which analyzes social efficiency by evaluating the water demand- and population-weighted change in water stress resulting from each transfer. In addition, a document-based qualitative analysis grounded in hydrosocial theory was conducted to explore governance narratives, trade-offs, and regional impacts.

**Results:**

Findings reveal substantial variation in both hydrological and social efficiency across the three IBTs. The Melen system transfer from Düzce demonstrates a relatively high efficiency in relieving Istanbul’s water stress with moderate ecological cost. In contrast, transfers from the Istranca system in Tekirdağ, and Kırklareli exhibit lower natural and social efficiency, suggesting a disproportionate burden on already water-stressed source regions. The qualitative assessment highlights that these transfers, while effective in meeting Istanbul’s supply needs, often reinforce centralized, supply-driven governance models and overlook the socio-environmental impacts on donor regions. Recurring droughts, reduced streamflow, and competition with local agricultural needs further exacerbate these tensions.

**Conclusions:**

The study demonstrates that water transfers can create uneven outcomes between donor and recipient regions, particularly when hydrological limitations and social vulnerabilities are not explicitly addressed in planning. While large-scale transfers may appear effective in securing urban water supply, they may also deepen regional inequalities and environmental risks. The findings call for a shift toward integrated and adaptive water governance models that consider long-term hydrological sustainability, ecosystem health, and inter-regional equity. For cities like Istanbul, this means rethinking reliance on external water sources and investing in demand management, local resilience, and participatory planning frameworks.

## Introduction

The world is facing a convergence of interlinked environmental and social challenges, including water scarcity, food insecurity, climate variability, biodiversity loss, and pollution, that are exacerbated by and, in turn, intensify the impacts of climate change. As global populations grow and economic activity expands, pressures on finite natural resources increase, particularly in urban areas, which are projected to house nearly 70% of the global population by 2050 (McDonald et al., 2020). Cities, as centers of economic and political power, are increasingly exposed to water-related risks due to escalating demand, rapid urbanization, and climatic stress. These dynamics are particularly evident in megacities that rely on distant water sources to meet growing demand, such as Istanbul, Türkiye.

Water security, defined broadly to include access, quality, reliability, and sustainability, has become a critical concern for urban areas (Cook & Bakker, 2012; Sivri et al., 2017; Keys, Wang-Erlandsson & Gordon, 2018). Currently, many of the world’s megacities (urban areas with populations over 10 million) are situated in regions facing some level of water scarcity, where demand either seasonally or structurally exceeds supply (He et al., 2021). Urban water demand is projected to increase by 80% by 2050 due to population growth, in migration, and changes in socio-economic development (United Nations, 2023). At the same time, climate change is expected to alter the spatial and temporal distribution of water resources, further complicating water management and planning (He et al., 2021; Caretta et al., 2022).

The prevailing response to urban water stress has been the pursuit of supply-side solutions, notably large-scale infrastructure such as inter-basin water transfers (IBTs). IBTs involve the redirection of water from donor basins, often rural and ecologically sensitive, to urban centers. While they can temporarily relieve supply deficits, IBTs are often associated with ecological degradation, socio economic displacement, and long-term path dependency, wherein increased supply induces greater demand and continued reliance on engineered solutions (Islar & Boda, 2014; Di Baldassarre et al., 2018; Rollason, Sinha & Bracken, 2021; Faundez et al., 2022). These dynamics often obscure demand-side alternatives and generate uneven trade-offs between donor and recipient regions.

Such dynamics are particularly evident in rapidly growing megacities that rely on water transfers to reconcile rising demand with geographically constrained supply. While a growing body of literature has examined urban vulnerability to climate and demographic pressures, less attention has been paid to the regional water availability, water stress conditions, and distributional impacts between donor and receiving basins, particularly in contexts where governance responsibilities are fragmented across jurisdictions. In Türkiye, water governance responsibilities are distributed across multiple institutions, including the State Hydraulic Works (DSI), metropolitan water utilities, and provincial authorities, creating coordination challenges in managing inter-basin transfers.

This study examines these dynamics through the case of Istanbul, Türkiye, a megacity that has become increasingly dependent on large-scale water transfers to secure its water supply. To address this gap, this study adopts a socio-hydrological lens to examine water security in Istanbul and its connected supply basins. Using a combination of a quantitative water security indices and qualitative document analysis, the study evaluates how large-scale water transfers affect the ability of the metropolitan water supply system to maintain reliable water availability under growing demand and climatic variability systems.

This approach aligns with calls from the socio-hydrology literature for integrated, multi-scalar assessments that account for human-water interactions, institutional dynamics, and long-term sustainability (Sivapalan, Savenije & Bloschl, 2012; Haeffner et al., 2021). By focusing on both Istanbul and its donor basins, this study contributes to ongoing debates on urban water security, inter-basin transfers, and water justice, and offers insights relevant to other megacities facing similar supply-side dependencies. While several studies have assessed Istanbul’s vulnerability to climate and demographic pressures (Daloğlu Çetinkaya et al., 2022; İlhan, 2022; Burak, Bilge & Ülker, 2021; Savun et al., 2021; Bakırcı, 2016; Islar & Boda, 2014; Akalın, Mertoğlu & Ertürk, 2025), research on the regional water security impacts of inter-basin dependencies remains limited, particularly in the less studied water-exporting provinces. To address this gap, this study adopts a socio-hydrological perspective to examine water security dynamics between Istanbul and its donor basins. Using quantitative indicators of natural and social transfer efficiency alongside qualitative document analysis, this study evaluates how large-scale water transfers redistribute water availability, alter regional water stress conditions, and generate governance challenges across jurisdictions. Particular attention is given to the implications for donor basins, where water withdrawals may affect local water availability, ecological conditions, and development priorities.

## Background

This section provides historical, institutional, and infrastructural context for Istanbul’s contemporary water system, situating current large-scale water transfers within the city’s long-term water provisioning trajectory.

Water has long played a central role in the urban development of Istanbul, historically known as Byzantium and later Constantinople. As the capital of the Roman, Byzantine, and Ottoman Empires over more than sixteen centuries, the city repeatedly outgrew its local water resources due to continuous population growth and limited freshwater availability (Ozis, Alkan & Ozdemir, 2020). Early settlements relied on wells, cisterns, and nearby streams, however as the city expanded, increasingly sophisticated and large-scale water infrastructure such as long-distance aqueduct systems became necessary. Roman-era interbasin water transfers from the Istranca Mountains were complemented by large cisterns which buffered seasonable variability (Ward, Smith & Crapper, 2020; Peker, 2023). After the Ottoman conquest in 1453, the city’s water network became more centralized and technically advanced. Further modernization occurred in the 19th century with the introduction of foreign-operated water utilities, followed by a shift toward public control in the early 20th century. Key institutional milestones included the establishment of Istanbul Water Administration (Istanbul Su İdaresi, ISI) in the 1930s and later the Istanbul Water and Sewerage Administration (Istanbul Su ve Kanalizasyon İdaresi, ISKI) in 1981, which consolidated responsibility for water supply and wastewater services at the metropolitan scale (Dinçkal, 2008; Cinar, 2009).

Despite these institutional developments, rapid urbanization, rural-to-urban migration, and industrialization throughout the second half of the 20th century sharply intensified water demand. Until the mid-1990s, Istanbul’s water supply was sourced almost entirely from within its municipal boundaries, relying on reservoirs such as Ömerli, Terkos, Elmalı, Darlık, and Büyükçekmece, as well as wells and the Yeşilvadi Regulator, which withdraws water without storage (ISKI, 2021; Akalın, Mertoğlu & Ertürk, 2025, Table 1). However, recurring droughts in the 1980s and 1990s, combined with sustained population growth, exposed the limits of these local sources and prompted a structural shift toward large-scale water transfers.

**Table 1:** Drinking water resources of Istanbul, adapted from ISKI (2025, 2025).

**Drinking water resource**	**Location**	**Annual yield capacity (Million m**^3^)	**Raw water abstracted in 2023 (Million m^3^)**	**Opening year**
*Resources within Istanbul’s borders*				
Ömerli Dam	Çekmeköy, Istanbul	220	24.3	1972
Darlık Dam	Şile, Istanbul	97	51.2	1989
Elmalı1 and 2 Dams	Beykoz, Istanbul	15	8.4	1893–1950
Terkos Dam	Arnavutköy, Istanbul	142	90.6	1883
Alibey Dam	Sultangazi, Istanbul	36	24.5	1972
Büyükçekmece Dam	Büyükçekmece, Istanbul	100	54.6	1989
Sazlıdere Dam	Küçükçekmece, Istanbul	55	40.8	1998
Yeşilçay System (İsaköy and Sungurlu Regulators)	Şile, Istanbul	145	158.6	2004
Yeşilvadi Regulator	Şile, Istanbul	10	3.9	1992
European Wells	Multiple locations	30	22.5	Various
*Inter-basin water transfers (IBTs)*				
Istrancalar Dams (considered as a whole IBT in Tekirdağ)		75	34.3	1995–1997
− Düzdere	Çatalca, Istanbul			
− Kuzuludere	Çatalca, Istanbul			
− Büyükdere	Çatalca, Istanbul			
− Elmalıdere	Tekirdağ			
– Sultanbahçedere	Tekirdağ			
Kazandere Dam	Kırklareli	100	25.5	1997
Pabuçdere Dam	Kırklareli	60	16.1	2000
Melen Regulators	Düzce	650	590	2007, 2014, 2023
**Total**		**1,795 Mm^3^/Year**	**1,145 Mm** ^**3**^ **/Year**	

Between 1995 and 2000, new reservoirs were developed in neighboring provinces to the west, namely the Istrancalar, Kazandere, and Pabuçdere systems (collectively the “Istranca system”), located approximately 150 km from Istanbul in Kırklareli and Tekirdağ. This expansion was followed by the implementation of Istanbul’s largest and most strategically significant transfer project, the Melen system, which diverts water from the Great Melen River in Düzce, approximately 180 km east of the city. Implemented in two main phases in 2007 and 2014, the Melen system substantially increased the city’s supply capacity (Ozturk et al., 2013; ISKI, 2021). In 2023, the system’s transfer capacity was further expanded by an additional pumping station, adding 75 million m^3^/year to meet rising demand driven by population growth and migration (ISKI, 2023). These external transfers were complemented by the development of additional in-city infrastructure, including the Sazlıdere Dam (1998), the Yeşilçay Regulator (2004), and upgrades to existing reservoirs (ISKI, 2005).

As of 2023, over 98% of Istanbul’s water demand for domestic and industrial use was met through surface water resources drawn from eleven separate watersheds (ISKI, 2023, Fig. 1). Groundwater was once a significant resource for the city but the reliance on surface water increased as populations increased and residential development led to water quality challenges (Istanbul Master Plan Consortium, 1999; ISKI, 2021). These resources are managed through a complex system of fourteen reservoirs and interlinked transfer schemes operated by ISKI, a municipally affiliated yet administratively autonomous utility. ISKI works in coordination with the 14th Regional Directorate of the State Hydraulic Works (Devlet Su İşleri, n.d.; Devlet Su İşleri, n.d.), which continues to play a central role in basin-level planning and large-scale water infrastructure development.

**Figure 1:**
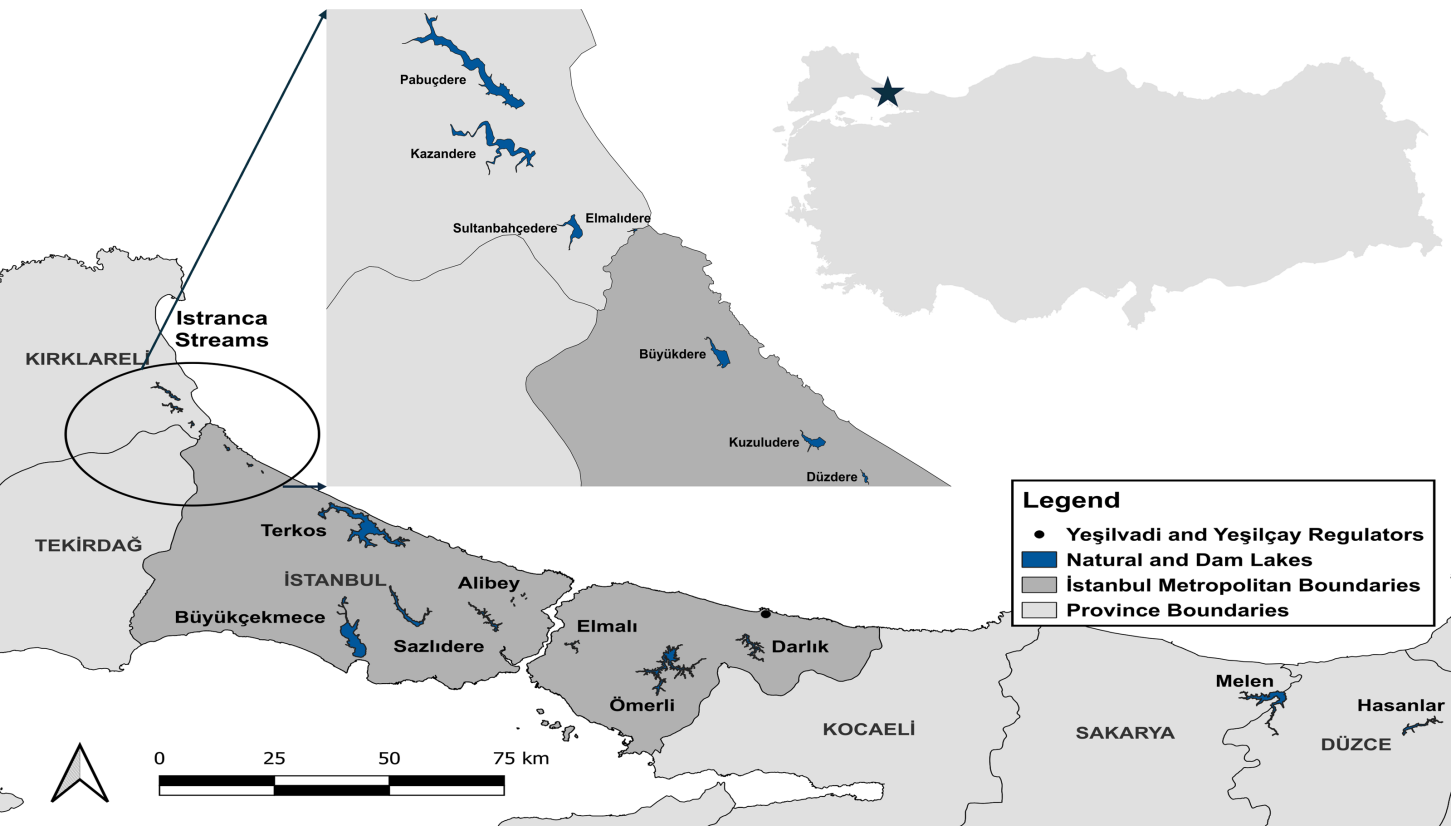
Water resources that supply Istanbul with drinking water. Blue areas denote reservoirs and dark grey area denotes the Istanbul Metropolitan boundaries. Istanbul’s water resources that are outside of the city’s boundaries are the Melen system to the east and the Istranca system to the west.

The spatial configuration of Istanbul’s water system further illustrates the challenges of inter-basin dependency. Approximately 62% of the city’s annual water yield is located on the Asian side of the metropolitan area, while roughly 60% of the population resides on the European side (ISKI, 2025). This imbalance necessitates a fully integrated distribution network, including interconnected reservoirs, underwater pipelines crossing the Bosphorus, and 24 drinking water treatment plants with a combined capacity exceeding 4.9 million m^3^ per day (ISKI, 2024; Van Leeuwen & Sjerps, 2016). Current challenges include continued rising water demand in Istanbul with a projected population increase to 21.3 million by 2050 (IBB, 2021) and declining local water resource yields caused by increased urbanization, unplanned settlements around water resources, and climate change. The water-exporting provinces also face increased water demand and impacts to water availability as a result of climate change (Varty Bayar, 2023; Aktaş, 2014).

Istanbul therefore represents a particularly suitable case for examining inter-basin water transfers through an integrated quantitative and qualitative lens. The city’s long-standing reliance on remote water sources reflects broader global tensions between urban water demand, geographic constraints, and multi-jurisdictional governance. Despite its significance as Türkiye’s largest city and a critical node in national water infrastructure, relatively few studies systematically assess how Istanbul’s inter-basin water transfers affect water-exporting regions. By situating contemporary transfers within their historical, institutional, and infrastructural context, this study provides a foundation for evaluating the broader socio-hydrological and governance implications of urban water dependency on water sources outside of the city’s boundaries.

## Materials & Methods

This study adopts a socio-hydrological perspective as its theoretical framework to examine the interactions between water availability, infrastructure, and societal responses in large-scale water transfers. Within this framework, the research employs a mixed-methods design and an in-depth case study of the Istanbul metropolitan water supply system and its donor basins.

The methodological design combines quantitative index-based analysis with qualitative document analysis to balance comparability and measurability with institutional and governance context. Quantitative analysis draws on indices to evaluate the natural and social efficiency of IBTs (Duan et al., 2022), while qualitative document and discourse analysis provides context on governance, policy narratives, and equity concerns. Together, these methods enable a holistic understanding of coupled human-water interactions and the multifaceted implications of large-scale water transfers, a central concern of socio-hydrological research. This integrated approach is particularly suited to cases such as Istanbul, where data availability enables quantitative comparison across basins, but socio-political dimensions must be explored through qualitative evidence. Socio-hydrology conceptualizes water systems as coupled human–water systems characterized by feedbacks between hydrological processes, infrastructure development, and societal responses (Sivapalan, Savenije & Bloschl, 2012; Di Baldassarre et al , 2019). In this study, the socio-hydrological perspective is used as a conceptual framework to analyze how water transfers redistribute water availability and reshape water security across both donor and receiving regions.

To operationalize this perspective, the study combines quantitative indices that measure the redistribution of water resources and changes in water stress with qualitative document analysis that examines governance structures, policy narratives, and equity concerns surrounding the transfers. While socio- hydrology often relies on quantitative modelling of human–water feedbacks, previous research has noted limitations in representing institutional capacity, political dynamics, and distributional equity within purely quantitative frameworks (Wesselink, Kooy & Warner, 2016; Carr et al., 2022; Xia, Dong & Zou, 2022). For this reason, the present study complements index-based analysis with qualitative evidence to better capture governance and socio-political dimensions of large-scale water transfers that cannot be represented through hydrological indicators alone.

### Indices to measure natural and social efficiency

Duan et al. (2022) have developed two efficiency indices to assess water transfer efficiency, based on the assumption that an increase or decrease in water availability as a result of a water transfer would respectively lead to a positive or negative impact to regional water supply and its efficiency. Here, efficiency is evaluated by weighing the benefit against the cost (Colby, 1990, as cited by Duan et al., 2022). The Natural Efficiency Index (DIO, Difference in transfer-In and transfer-Out), measures “natural efficiency” by measuring the difference between the transfer-in ratio and transfer-out ratio between the receiving and exporting regions (Eq. 1). The Stress Relief Index (SRI) measures the “social efficiency” of how overall water stress in the water-exporting or receiving watersheds is impacted by the water transfer (Eq. 2). The selection of these indices is motivated by their ability to provide a transparent and comparable assessment of inter-basin water transfers across donor and recipient regions using publicly available data.

For the purposes of this study, the receiving region is Istanbul, whose integrated water distribution network means no one district is receiving water from a single watershed, and the exporting regions are Düzce for the Melen system transfer and Tekirdağ and Kırklareli for the Istranca system transfer. The majority of the Melen watershed is in the administrative bounds of Düzce and bordering provinces such as Sakarya do not rely on water resources within the Melen watershed, which is why only Düzce has been considered. The streams within the Istranca system drain directly into the Black Sea without crossing additional provincial borders. Defining the study area using these administrative borders was necessary for data availability constraints and aligns with Duan et al. (2022) who recommend looking beyond the individual watersheds of the transfers to assess efficiency upstream and downstream since water transfers can impact a much larger area. These indices are particularly suitable for the Istanbul case, where long-term, basin-level socio-hydrological data are limited, but annual hydrological, demand, and population statistics are consistently reported by public institutions.

The Natural Efficiency Index (DIO) measures the relative change in water availability between receiving and exporting regions resulting from an IBT by comparing the share of transferred water relative to regional accessible “natural” renewable freshwater availability in both the receiving and exporting regions (Duan et al., 2022). It is calculated as the difference between the transfer-in ratio (TI), the proportion of transferred water to renewable freshwater availability in the receiving basin and the transfer-out ratio (TO), the same proportion in the exporting basin. Thus, the index reflects the contrast in relative water availability between two regions rather than the absolute sustainability of withdrawals from the donor basin. This difference is expressed as:

(1) \begin{eqnarray*}DIO=TI-TO= \frac{T}{T{F}_{m} \left( r \right) } - \frac{T}{T{F}_{m} \left( e \right) } \end{eqnarray*}

where T is the volume of water transferred (Mm^3^/year), T*F*_m_(r) is the renewable freshwater availability in the receiving region (Mm^3^/year), and T*F*_m_(e) is that of the exporting region (Mm^3^/year). A positive DIO indicates the transferred volume represents a relatively larger share of water availability in the receiving region than in the exporting region, suggesting a comparative hydrological advantage for the transfer. Conversely, a negative value indicates that the withdrawal places a relatively greater proportional burden on the exporting region’s water resources. This index relies on annual estimates of renewable freshwater availability for the exporting provinces based on surface water and groundwater data reported by the State Hydraulic Works (DSI) and provincial environmental status reports. It should therefore be interpreted as an indicator of relative water allocation efficiency rather than a comprehensive measure of donor basin sustainability. As such, it does not capture seasonal variability, short-term hydrological extremes, unreported abstractions, or climate-induced intra-annual changes. In addition, the indicator does not explicitly account for ecological thresholds or cumulative hydrological stress associated with large transfer volumes. These limitations reflect data availability constraints rather than the theoretical structure of the index.

The Stress Relief Index (SRI) measures social efficiency by evaluating the population-weighted relief in water stress attributable to the water transfers, thereby enabling the assessment of the distributional impacts of water transfers on human water security. The index is calculated for the receiving and exporting regions separately and defined as:

(2) \begin{eqnarray*}SRI= \frac{{\lambda }_{i}x{P}_{i}x(-\Delta W{S}_{i})}{T} \end{eqnarray*}

where Δ*WS*_*i*_ represents the change in water stress in the studied regions, P_*i*_ is the affected population, *λ*_*i*_ is a weighting factor reflecting regional sensitivity or policy priority, and T is the volume of water transferred. Following Duan et al. (2022), the weighting factor of *λ*_*i*_ is set to a constant value of 1 to ensure consistent comparisons between the provinces that experience broadly similar climate change impacts. The SRI should therefore be interpreted as a comparative indicator that evaluates the distributional benefits of inter-basin water transfers across regions rather than as a physical quantity with strict dimensional meaning. In this formulation, the normalization occurs through the water stress component (ΔWS), which reflects relative changes in water demand pressure with respect to available freshwater resources. Water stress (WS) is defined as the ratio of off-stream water demand to renewable freshwater availability and is therefore a dimensionless indicator. Consequently, the change in water stress (ΔWS) is also dimensionless. The change in water stress ΔWS_*i*_ is calculated for both the receiving and exporting regions separately, as:

(3) \begin{eqnarray*}\Delta WS=WD\times \left( \frac{1}{T{F}_{ct}} - \frac{1}{T{F}_{c}} \right) \end{eqnarray*}

where WD represents water consumption in the receiving and exporting regions, respectively, and T*F*_*ct*_, T*F*_*c*_ are the renewable freshwater availability in the exporting and receiving regions. All variables in the calculation are expressed in consistent volumetric units (Mm^3^/year), ensuring comparability of the resulting values across regions and time periods.

The SRI assumes water demand and population are adequately captured through official annual statistics, although industrial withdrawals or unregistered water use may not always be fully captured due to limited disaggregated data availability. Furthermore, population data are assumed to represent water service areas, which may differ from hydrological basin boundaries. Both indices offer complementary insights into the effectiveness of water transfers. Divergences between DIO and SRI (*i.e.,* DIO x SRI < 0) signal trade-offs between hydrological and social outcomes. High-volume transfers may reduce efficiency in both indices, by increasing pressure on exporting basins while generating diminishing marginal benefits for receiving regions approaching demand saturation. In this study, index results are interpreted in conjunction with qualitative document analysis to contextualize efficiency outcomes within broader governance, institutional, and socio-political dynamics that cannot be directly captured by the indices alone, an approach commonly adopted in socio-hydrology studies (Mostert, 2018; Haeffner et al., 2021).

### Data requirements

The analysis relies on publicly available hydrological, demographic, and water use data to calculate the indices. Variables include water transfer volumes, freshwater availability, municipal and agricultural consumption, and population statistics for Istanbul, Düzce, Tekirdağ, and Kırklareli. Data were sourced from the Istanbul Water and Sewerage Administration (ISKI), State Hydraulic Works (DSI), Turkish Statistical Institute (TUIK), and provincial environmental reports. Agricultural data were cross-validated with provincial reports. Some interannual estimates were interpolated where data gaps existed (*e.g.*, pre 2010 values for Melen, data was unavailable for all regions in some years) (Table 2). For the full dataset, see Appendix SA.

**Table 2:** Data requirements.


Study area	Istanbul, Düzce, Tekirdağ, Kırklareli
Study boundaries	Each province’s provincial boundaries (Tekirdağ and Kırklareli as one region)
Inter-basin water transfers (IBTs)	Melen Watershed (Düzce) Istranca Streams [Istrancalar, Kazandere, and Pabuçdere] (Tekirdağ and Kırklareli)
Timeline^*^	Melen: 2008–2023 Istranca Streams: 2000–2023
Timestep	1 year
Data sources	Provincial Environmental Status Reports, ISKI Annual Reports, TUIK Regional Statistics, DSI, TESKI

Efficiency metric	Abbreviation	Explanation	Unit
Transfer-in ratio	TI	Proportion of transferred water to renewable freshwater availability in the receiving basin	n/a
Transfer-out ratio	TO	Proportion of transferred water to renewable freshwater availability in the exporting basin	n/a
Difference between TI and TO	DIO	TI minus TO	n/a
Stress Relief Index	SRI	Population-weighted relief in water stress attributable to the IBT	thousand people . Mm^−3^

**Data needed**	**Symbol**	**Unit**	**Source (2000–2023)**
*Natural efficiency*			
Transfer magnitude for each IBT	T	Mm^3^ /year	ISKI
Freshwater availability for each province (ground and surface water)	TFm(r) for receiving TFm(e) for exporting	Mm^3^/year	Regional Environmental Status Reports, DSI
*Social efficiency (Stress Relief Index)*			
Transfer magnitude for each IBTs	T	Mm^3^/year	ISKI
Population of each region	P	thousand people	TUIK
Weighing factor based on water management priorities	*λ*	n/a	Duan et al. (2022)
Change in water stress due to IBT	ΔWS	n/a	Calculated in the analysis
Regional water use (residential, industrial, commercial, agricultural)	WD	Mm^3^/year	TUIK Regional statistics, ISKI, TESKÍ, Regional Environmental Status Reports, DSI
Freshwater availability for each province (ground and surface water)	TFct for exporting TFc for receiving	Mm^3^/year	Regional Environmental Status Reports, DSI

**Notes.**

* 2023 values are used in place of 2024 because the 2024 data is not available.

### Document analysis

This study uses document analysis to examine qualitative data from academic literature, government and NGO reports, and planning documents published by the Istanbul Metropolitan Municipality (İstanbul Büyükşehir Belediyesi, IBB), ISKI, DSI, provincial governments, the Istanbul Master Plan Consortium (IMC), and World Wildlife Fund (WWF). Following Bowen (2009), this approach allows for the identification of underlying themes and insights into governance and justice dimensions of water security and water transfers. Documents were selected based on relevance, credibility, and temporal coverage from 1994–2025 to account for the time period of the transfers beginning with the first phase of the Istranca system transfer in 1995. Analysis focused on how the water transfers to Istanbul are justified, debated, and contested across scales.

Documents were selected for their relevance to Istanbul’s water governance and the impacts of the water transfers. The analysis revealed four key themes: (1) environmental and socio-economic impacts of water transfers (2) the supply–demand cycle, where expanding supply drives further urban growth; (3) political framing and governance complexity arising from supply-side management priorities; and, (4) limited attention to the effects of water transfers on water-exporting provinces (Appendix SB). This component complements the quantitative indices by addressing dimensions not captured in efficiency metrics, such as procedural justice, political narratives, and power asymmetries, strengthening the socio-hydrological framing of the study.

## Results

### Quantitative analysis

**Figure 2:**
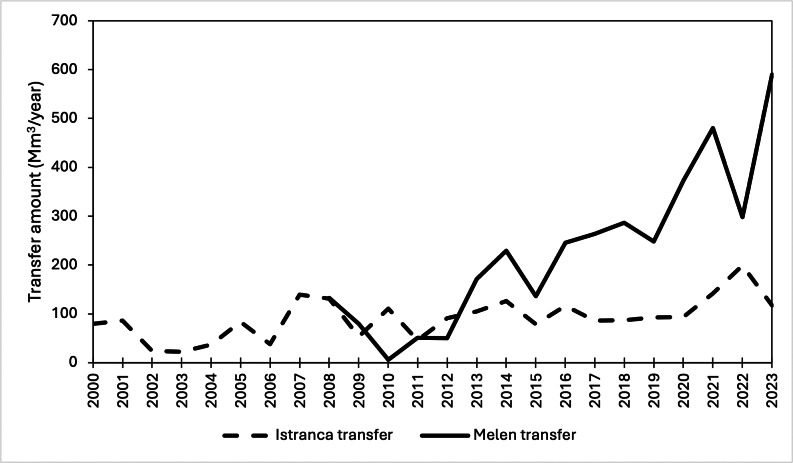
The transfer amounts from the Istranca and Melen IBTs to Istanbul from 2000–2023. The Melen IBT did not begin operating until 2008. Dashed lines for Istranca reservoirs and straight line for Melen river transfers.

The annual volumes of water transferred to Istanbul from the Melen and Istranca systems have steadily increased between 2000 and 2023, highlighting the city’s increasing reliance on water transfers to meet growing water demands (Fig. 2). While transfers from the Istranca system (Istrancalar, Kazandere, and Pabuçdere) have remained relatively stable with year-to-year fluctuations, the Melen system shows a marked upward trend, particularly after 2012, when major infrastructure expansions were completed. This shift reflects a strategic move toward more intensive use of the Great Melen River as a long-term water source for Istanbul.

**Figure 3:**
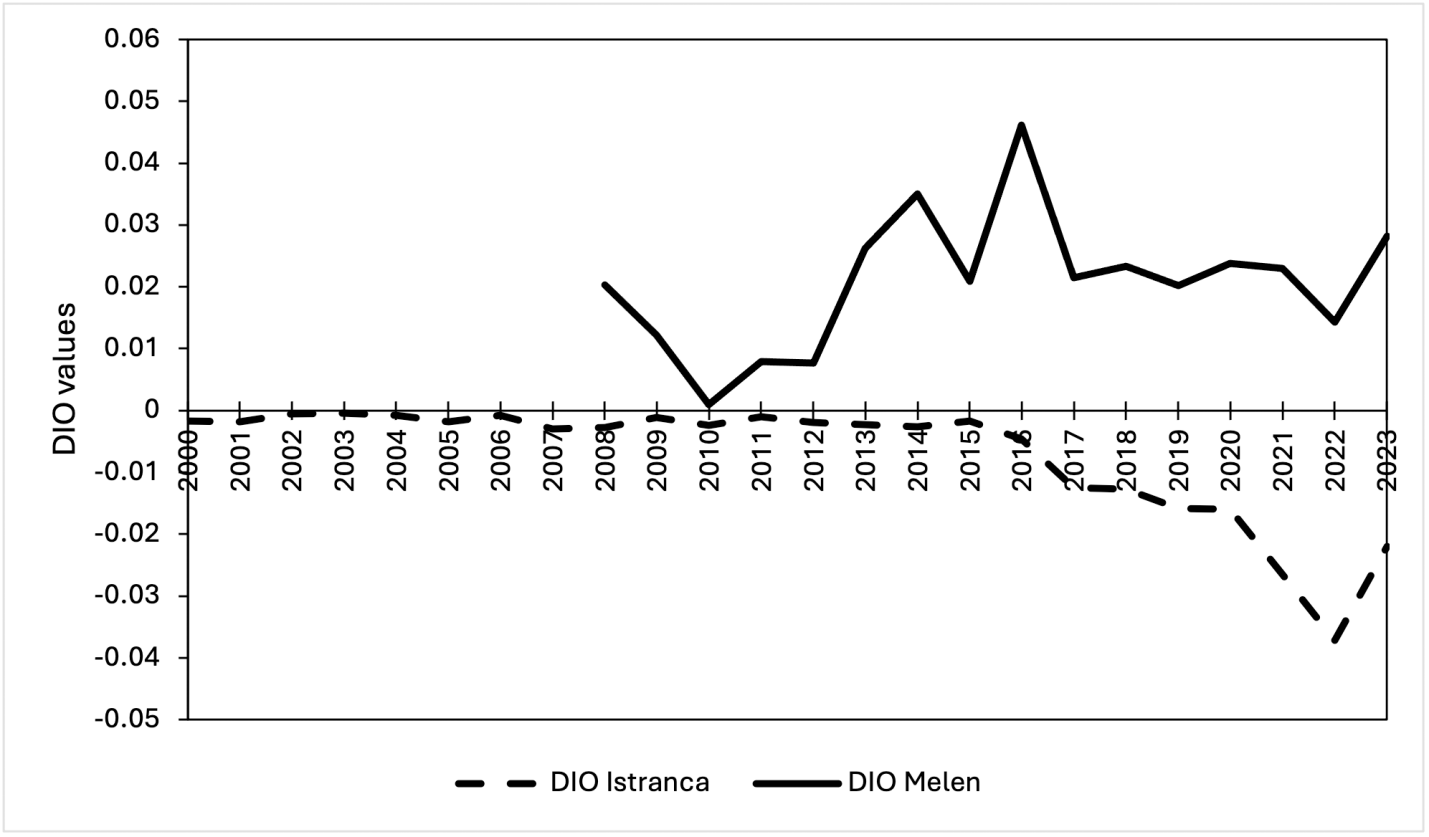
Temporal variations in natural efficiency (DIO) values for the Istranca and Melen transfers. Dashed lines for Istranca system and straight line for Melen river transfers. Transfers from the Melen River started in 2008.

The Natural Efficiency Index (DIO) evaluates whether water is transferred from regions of higher to lower availability, transfers in the opposite direction are considered inefficient and potentially harmful to the environment. However, it should be noted that the DIO indicator primarily reflects the relative contrast in water availability between the receiving and exporting regions; therefore, positive values should not be interpreted as unconditional evidence that large transfer volumes are hydrologically sustainable for the donor basin. In Istanbul’s case, the Melen system transfer shows slightly higher natural efficiency than the Istranca system, due to Düzce’s greater water potential (Fig. 3, Table 3). However, both transfer routes exhibit DIO values near zero, indicating neutral efficiency. As Duan et al. (2022) note, higher transfer volumes generally raise natural efficiency when water flows from a more water-abundant to a water-scarce region. This is evident in the Melen system transfer, where efficiency increased between 2012–2014 and 2021–2023 following increased transfer capacity infrastructure. Due to the Great Melen River, Düzce has higher water availability than Istanbul, natural efficiency for this transfer remains positive. Conversely, transfers from Tekirdağ and Kırklareli (Istrancalar, Kazandere, and Pabuçdere), which have lower water potential than Istanbul, yield negative values regardless of transfer volume. Between 2014 and 2023, 8%–34% of Düzce’s water availability was transferred annually, while 2%–16% was removed from Tekirdağ and Kırklareli’s availability since 2000. These withdrawal shares provide an additional perspective on the pressure imposed on the donor basins and should be interpreted alongside the DIO indicator to better assess the magnitude of hydrological stress associated with the transfers.

**Table 3:** Results of the water security indices for Istanbul’s water transfers.

Istranca Streams (Istrancalar, Kazandere, and Pabuçdere) - Tekirdağ and Kırklareli												
*2000–2011*												
	**2023**	**2022**	**2021**	**2020**	**2019**	**2018**	**2017**	**2016**	**2015**	**2014**	**2013**	**2012**
Transfer amount (million m^3^/year)												
Istranca	117.53	199.01	141.95	93.29	92.989	86.86	85.95	116.09	78.75	126.15	104.64	90.92
Natural efficiency (DIO, transfer-in –transfer-out)												
Istranca	−0.0219	−0.0372	−0.0265	−0.0159	−0.0159	−0.0127	−0.0125	−0.0046	−0.0017	−0.0027	−0.0022	−0.0019
Social efficiency (SRI, thousand people x Mm^−3^)												
Istanbul	−27.82	−16.49	−22.41	−30.39	−30.26	−26.36	−26.05	−5.100	−3.84	−2.31	−2.63	−3.03
Tekirdağ and Kırklareli	−0.7491	−0.4329	−0.5669	−0.7733	−0.7493	−0.6401	−0.6218	−0.1191	−0.0875	−0.0515	−0.0633	−0.0713

*2000–2011*												
	**2011**	**2010**	**2009**	**2008**	**2007**	**2006**	**2005**	**2004**	**2003**	**2002**	**2001**	**2000**
Transfer amount (million m^3^/year)												
Istranca	45.93	110.45	53.79	131.16	139.66	37.53	83.944	36.9	22.04	23.84	86.46	79.34
Natural efficiency (DIO, transfer-in –transfer-out)												
Istranca	−0.0009	−0.0024	−0.0012	−0.0028	−0.0029	−0.0008	−0.0018	−0.0008	−0.0005	−0.0005	−0.0019	−0.0017
Social efficiency (SRI, thousand people x Mm^−3^)												
Istanbul	−5.22	−1.99	−3.71	−1.49	−1.37	−5.09	−2.10	−4.89	−7.46	−6.69	−1.99	−2.03
Tekirdağ and Kırklareli	−0.1383	−0.0556	−0.1140	−0.0469	−0.0430	−0.1601	−0.0677	−0.1459	−0.2396	−0.2155	−0.0578	−0.0613

Melen Regulators – Düzce																
2008–2023																
**2023**	**2022**	**2021**	**2020**	**2019**	**2018**	**2017**	**2016**	**2015**	**2014**	**2013**	**2012**	**2011**	**2010**	**2009**	**2008**
Transfer amount (million m^3^ /year)																
Melen	590.01	298.17	480.52	372.31	247.88	286.72	263.33	245.44	136.2	228.97	171.18	50.43	51.31	5.89	79.65	132.51
Natural efficiency (DIO, transfer-in–transfer-out)																
Melen	0.0282	0.0142	0.0229	0.0238	0.0202	0.0234	0.0214	0.0462	0.0209	0.0351	0.0262	0.0077	0.0079	0.0009	0.0122	0.0203
Social efficiency (SRI, thousand people x Mm^−3^)																
Istanbul	1.42	2.81	1.69	2.85	5.41	4.46	4.74	11.56	15.91	9.15	11.52	39.17	33.49	268.15	17.97	10.54
Düzce	0.0015	0.0029	0.0017	0.0027	0.0049	0.0039	0.0039	0.0088	0.0125	0.0076	0.0101	0.0326	0.0327	0.2900	0.0219	0.0129

**Figure 4:**
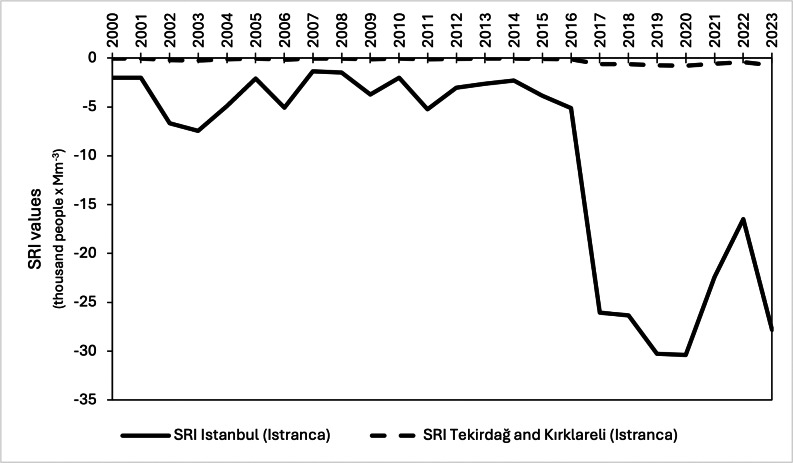
Temporal variations in Stress Relief Index (SRI) values for the Istranca transfer to Istanbul from Tekirdağ and Kırklareli The straight line denotes the SRI values for Istanbul (receiving region) and the dashed line denotes the SRI values for Tekirdağ and Kırklareli (donor region).

**Figure 5:**
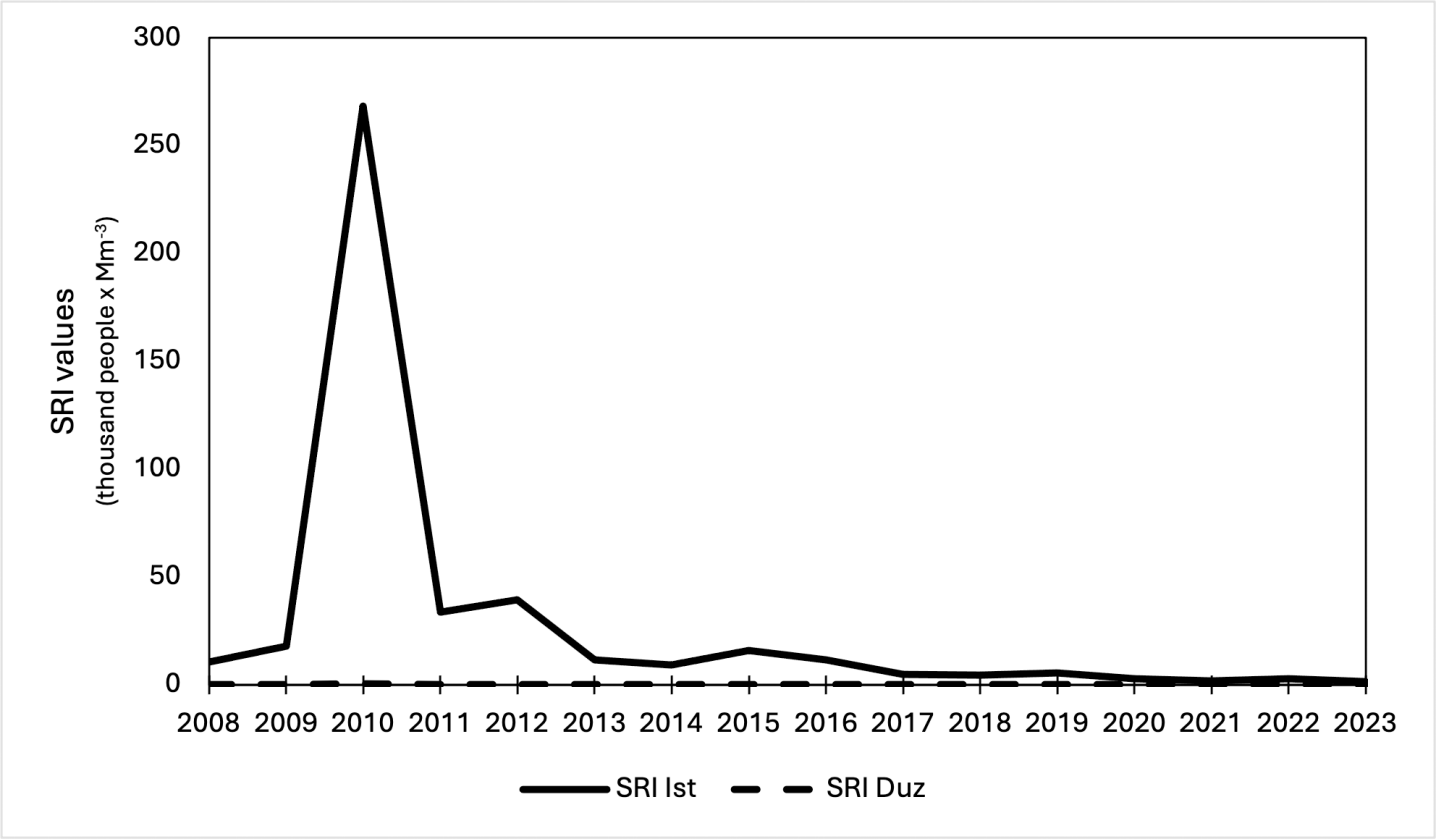
Temporal variations in Stress Relief Index (SRI) values for the Melen transfer: Istanbul and Düzce. Straight line denotes SRI for Istanbul (Melen) and dashed line denotes SRI for Düzce (Melen).

The SRI values, which consider population and water demand, for the Istranca system transfers yield negative values for both Istanbul and the donor provinces (Tekirdağ and Kırklareli), indicating inefficiency in alleviating water scarcity. This stems from Istanbul having a higher water availability than the exporting provinces. According to Duan et al. (2022), such conditions render the transfers socially inefficient for both regions. The more pronounced negative value in 2020 reflects a sharp increase in Istanbul’s water potential between 2016 and 2018, which lowered local water stress and widened the gap with the donor provinces. While SRI values for Tekirdağ and Kırklareli remain negative, they are closer to zero, as their lower water demand and population moderate the overall impact (Fig. 4). The transfer from the Melen watershed in Düzce shows a positive social SRI for Istanbul, reflecting increased water availability. However, efficiency declines as transfer volumes rise, consistent with Duan et al. (2022), who found that smaller transfers are more efficient under the SRI, benefiting recipients without significantly depleting donor resources. Higher SRI values in 2010–2012 correspond to lower transfer volumes during drought years, which paradoxically improved efficiency by reducing strain on both basins. In contrast, the lowest SRI value in 2023 reflects Istanbul’s growing dependence on the Melen system, which peaked at 590 Mm^3^/year following the completion of a third pumping station (Fig. 5). For Düzce, social efficiency values remain slightly negative and worsen with higher transfer volumes, though the impact is moderated by the province’s high water potential and low population, resulting in index outcomes comparable to Tekirdağ and Kırklareli despite larger exports.

### Qualitative analysis

Document analysis revealed four key themes: (1) environmental and socio-economic impacts of water transfers; (2) the supply–demand cycle, where increasing supply drives further urban growth; (3) political framing and governance challenges arising from supply-side management priorities; and (4) limited attention to the effects of transfers on water-exporting provinces (Appendix SB). A review of academic studies, government and NGO reports, and media sources highlights that water transfers effect not only water availability but also reinforce spatial and social inequalities between Istanbul and donor basins. In addition, Istanbul’s future water scarcity risks are projected to intensify by 2030–2050 (Daloğlu Çetinkaya et al., 2022; Akalın, Mertoğlu & Ertürk, 2025) or 2060 (Burak, Bilge & Ülker, 2021) under different climate change and water supply scenarios. Governance issues, particularly fragmented institutional authority and politically framed justifications for supply augmentation, play a central role in justifying water transfers. These findings support critiques of socio-hydrology, which emphasize the need to go beyond modeling to account for institutional, historical, and cultural drivers shaping human-water interactions.

### Environmental and socio-economic impacts

Large-scale interbasin water transfers and dam infrastructure have significantly altered hydrological regimes and ecosystems in Türkiye, particularly in regions supplying water to Istanbul (Burak, Bilge & Ülker, 2021). McDonald et al. (2020) and McGrane (2016) note that such infrastructure interrupts natural river flows and impacts rainfall capture and storage. National-level data for the country show a 16% decrease in surface water flows between 1995 and 2002 and rising river temperatures (∼0.2 °C annually), trends attributed primarily to increased water abstraction and land use change rather than solely to climate change (Aktaş, 2014). These trends highlight how Istanbul’s water demand is embedded in broader landscape transformations. Local overexploitation from Istanbul’s water sources has increased vulnerability to droughts (Istanbul Master Plan Consortium, 1999) and concerns about reduced flow in the Melen and Istranca systems (Islar & Boda, 2014; İlhan, 2022). While current abstractions remain within the safe yields defined by the ISKI Master Plan (Istanbul Master Plan Consortium, 1999), future projection scenarios raise significant concerns. For instance, if the Melen system transfer reaches 1,077 million m^3^/year as planned, Düzce could lose 62% of its freshwater resource capacity (ISKI, 2021; Düzce Valiliği , 2012-2023), highlighting the need for further evaluation. Water transfers can greatly impact the ecology of donor watersheds. Reduced downstream flows due to the Istranca systems could severely affect wetlands and marsh habitats, especially during dry years (Istanbul Master Plan Consortium, 1999; Karakaya, Evrendilek & Gonenc, 2014). WWF Turkey (2012) identified 11 endemic plant species affected by Melen Dam, including *Cyclamen coum*, a Bern Convention-protected species. The Istranca system has altered upstream and downstream habitats and threatens the İğneada Longos forest in Kırklareli, an ecologically critical wetland hosting hundreds of plant and animal species, including those listed in Appendix SI of the Bern Convention (Karakaya, Evrendilek & Gonenc, 2014). These findings align with concerns raised by Gupta & vander Zaag (2008) regarding the disproportionate ecological burden IBTs place on donor basins.

Economically, IBTs tend to favor the receiving regions, such as Istanbul, by supporting growth and increasing national GDP, while imposing significant costs on water-exporting communities, including loss of land, livelihoods, and long-term economic opportunities (Faundez et al., 2022). For the Melen system project, 16 villages in Düzce and Sakarya were resettled, with four settlements in Sakarya’s Kocaali district fully submerged for dam construction (Islar & Boda, 2014; Bakırcı, 2016). Reports highlight prolonged compensation delays, with some residents waiting years or receiving no compensation at all (Islar & Boda, 2014). Due to delays in the resettlement process, many families were forced to leave their homes before suitable resettlement options were available, and displaced residents faced diminished economic prospects, dissatisfaction, and broken social bonds as a result of urban relocation (İlhan, 2025). The uncertainty caused by extended planning timelines further discouraged investment in the region and triggered early rural-to-urban migration (Islar & Boda, 2014; İlhan, 2025). Moreover, pollution controls implemented to protect Melen water sources restricted local industries, such as textile dyeing (Islar & Boda, 2014).

Similar patterns are observed in the Istranca system region, where land expropriation reduced agricultural incomes and pushed residents toward precarious livelihoods like seasonal tourism and fisheries, both of which were also affected by reduced water availability (İlhan, 2025; Acara, 2019). Those that were resettled in urban areas faced difficulties adapting to urban lifestyles, while those who remained near the Kazandere and Pabuçdere dams lost their water access and livestock grazing rights (İlhan, 2025). Communities near planned reservoirs have voiced strong opposition to anticipated land loss and economic disruption (Güvemli, 2017). Even with compensation, communities impacted by both transfers faced “lasting impoverishment and social fragmentation” as a result of the loss of traditional livelihoods and cultural bonds, structural barriers to alternative economic opportunities, and lack of long-term support for adaptation (İlhan, 2025, p. 89). These findings underscore the water justice issues associated with Istanbul’s water transfers, while the city continues to benefit, the burdens fall disproportionately on donor basins. As Bakırcı (2016) notes, affected communities experience emotional and economic harm from displacement, weak expropriation processes, and development uncertainty, factors that fuel rural-to-urban migration and reinforce Istanbul’s dominance in growth and resource demand.

### Supply–demand cycle

Research and document analysis highlight Istanbul’s increasing dependence on water transfers from outside of the city’s as both a response to and driver of continued urban expansion and rising water demand. The literature identifies a feedback loop, described as the “supply–demand effect” by Di Baldassarre et al. (2018), where increases in water supply stimulate further urban growth, ultimately deepening water scarcity. Historically, this pattern has justified the development of both local water infrastructure (*e.g.*, Büyükçekmece, Darlık, Sazlıdere Dams) and major transfers from the Melen and Istranca systems (ISKI, 2021). Despite recent declines in population growth rates, Istanbul remains the country’s largest city and continues to experience significant in-migration and speculative development pressures (IBB, 2018; IBB, 2021; Güneralp, Tezer & Albayrak, 2013; Doğruel & Doğruel, 2018). Istanbul’s population has also increased by 30% since 1980 and is projected to reach 21.3 million by 2050, leading to increased water stress and a continuation of the supply–demand cycle (TUIK, 2023a; TUIK, 2023b; IBB, 2021). Land use and cover changes have been dramatic: between 1980 and 2017, built-up areas increased by 183.5%, while forest cover declined from 56.1% to 49.6% (Cengiz, Atmis & Gormus, 2019). This unregulated growth not only impacts local water availability but degrades water quality due to impervious surfaces, wastewater discharge, and illegal sewer connections (McGrane, 2016; Burak, Bilge & Ülker, 2021; IBB, 2021). Reservoirs like Ömerli have seen declining water yield and increased pollution from surrounding unplanned settlements (Güneralp, Tezer & Albayrak, 2013; Saatci, 2013). Ömerli’s raw water supply dropped from 164 million m^3^/year in 2018 to just 24 million m^3^/year in 2023 (ISKI, 2018; ISKI, 2023). Urban runoff, industrial pollution, and road development further compromise water bodies, threatening ecological health and increasing treatment demands (İlhan, 2022; Bekiroğlu & Eker, 2011). Despite protective regulations such as the 1984 Drinking Water Basin Regulations enacted by ISKI, enforcement has been inconsistent (Saatci, 2013; Sözen et al., 2021). Several water sources, including Küçükçekmece Lake and many former urban wells have already been rendered unusable due to pollution and saltwater intrusion (Istanbul Master Plan Consortium, 1999; ISKI, 2021).

### Political framing and governance challenges

The third theme emerging from the document analysis centers on the political framing and governance challenges surrounding water transfers to Istanbul. These projects are often justified as responses to climate-induced droughts and generalized water scarcity, rather than the consequences of long-term urbanization and rising demand (Islar & Boda, 2014). Framing large scale water transfers like the Melen and Istranca systems as technical solutions to droughts masks their broader socio-political and environmental impacts. Official discourse continues to portray the Melen system as “insurance” for Istanbul’s water supply (Islar & Boda, 2014), despite evidence that climate events increasingly affect both donor and receiving basins (Karakaya, Evrendilek & Gonenc, 2014; Rollason, Sinha & Bracken, 2021; İlhan, 2025). The water from the Istranca streams was considered flowing “uselessly” into the Black Sea before being transferred to Istanbul (ISKİ, 1994), ignoring local uses. This is embedded in a more recent official regulation on water resources allocation by DSI which prioritizes potable domestic water supply before environmental flows or other sectors such as irrigation and industry (Official Gazette issue 30947 (10/12/2019) cited by Akalın, Mertoğlu & Ertürk 2025). Governance concerns are also growing: the overlapping responsibilities of ISKI, IBB, DSI, and various ministries create fragmented authority, hindering effective water management, enforcement of water protection zones, and pollution control (Istanbul Master Plan Consortium, 1999; ISKI, 2021). Urban expansion and illegal settlements in reservoir protection zones, along with weak enforcement capacity, exacerbate these issues (Saatci, 2013; Burak, Bilge & Ülker, 2021).

Additionally, the neoliberal turn in Turkish water governance since the 1980s has facilitated the gradual commodification and partial privatization of water resources. Legislation such as Laws No. 3096 and 3291, and later Law No. 4628, encouraged private sector involvement in water infrastructure, shifting DSI’s role from public service provider to market enabler (Harris & Islar, 2013; Islar, 2012; Conker, 2016). These reforms supported DSI’s transformation into a regulator facilitating large-scale hydropower and water transfer projects, while urban utilities like ISKI were restructured to operate on a profit-oriented model, setting water tariffs to ensure minimum financial returns (Cinar, 2009). This approach reflects broader trends in global water governance, where the state’s role transitions from direct provider to facilitator for private actors (Harris & Islar, 2013). While not all privatization initiatives succeeded, especially in the face of public resistance, this shift raises critical questions about equity, access, and justice in Istanbul’s water governance, particularly as rural and donor regions bear the brunt of supply-side solutions.

Nonetheless, there is evidence of coordinated efforts to improve Istanbul’s water security and increase demand-side management. The Elmalı reservoir, previously polluted, was restored following the 1993–94 drought and reintroduced into the city’s supply system (Saatci, 2013; ISKI, 2021). Recent efforts by ISKI include stream restoration, sediment removal, and removing illegal buildings around key reservoirs to protect water quality (ISKI, 2006-2024). Demand-side water management activities include reducing water losses within the system, encouraging responsible water consumption, and reuse management, such as through wastewater and rainwater treatment and reuse (ISKI, 2006-2024; Savun et al., 2021; IBB, 2021). Rainwater harvesting-oriented policies have also been included in national and municipal regulations (Peker, 2023). These actions suggest that while Istanbul’s urban expansion continues to strain water systems, coordinated management and policy enforcement can mitigate some of these impacts.

### Limited attention to the effects of water transfers on water-exporting provinces

Although many studies acknowledge concerns regarding Istanbul’s growing reliance on water transfers, considerably less attention has been paid to their implications for the water-exporting provinces. Existing research has largely focused on socio-economic dimensions of the transfers in donor regions (Islar & Boda, 2014; Bakırcı 2016; Acara, 2019; İlhan, 2021; İlhan, 2025), while only a limited number of studies have examined ecological impacts (WWF Turkey, 2012; Karakaya, Evrendilek & Gonenc, 2014). Beyond these contributions, systematic assessments of how the transfers affect the long-term water security of donor provinces remain scarce.

This gap is particularly critical given that populations and water demands in the donor provinces are also increasing, alongside efforts to expand local water supply systems. These regions face mounting pressures on water quantity and quality, driven by industrial development, agricultural intensification, and unplanned urbanization (Varty Bayar, 2023). The majority of Düzce’s domestic water supply comes from the Ugur Stream, which flows into Efteni Lake, the source of the Great Melen River, and also provides hydroelectricity and irrigation (Düzce Valiliği , 2012-2023). Another important source, the Hasanlar Dam is built on the Great Melen River and also provides domestic use, hydroelectricity, and irrigation (Düzce Valiliği , 2012-2023). For Kırklareli Valiliği (2012-2023) Kırklareli, the province mainly relies on surface water for domestic and agricultural water use, with the Kırklareli Dam providing 95% of domestic water needs. Kırklareli Valiliği (2021) Tekirdağ relies mostly on groundwater for domestic and industrial use, while surface water sources such as the Kayalıköy and Çokal Dams supply irrigation and some domestic water needs. Among the donor provinces, Tekirdağ stands out due to its relatively larger population, intensive industrial activity, and higher dependence on groundwater, which heightens vulnerability to pollution and overexploitation (Tekirdağ Valiliği, 2012-2020). Despite these emerging risks, the cumulative effects of exporting water to Istanbul on donor-basin resilience remain poorly understood. As highlighted by (Daloğlu Çetinkaya et al., 2022), there is a pressing need for further research that evaluates how inter-basin transfers reshape water security in neighboring regions and how decision-making authority, benefits, and burdens are distributed across connected basins.

## Discussion

While water security indices are useful for assessing the impacts of interbasin water transfers (IBTs), they often abstract away the socio-political, institutional, and historical processes shaping water use and decision making. Socio-hydrology addresses this limitation by highlighting how governance structures, political choices, and human behavior co-evolve with hydrological systems (Sivapalan, Savenije & Bloschl, 2012; Mostert, 2018; Haeffner et al., 2021), an insight reflected in our findings. To better capture these dimensions, document analysis was used alongside the quantitative index-based assessment. Drawing on this integrated approach, the Discussion advances three interrelated arguments: (i) efficiency equity trade-offs revealed by the indices, (ii) institutional lock-in and governance dynamics sustaining reliance on water transfers, and (iii) the justice implications and hydrosocial dynamics associated with these transfers.

Overall, the results indicate that Istanbul’s continued dependence on external water sources is shaped not only by hydrological constraints, but by governance dynamics and political framing. The near-zero and negative DIO and SRI values provide quantitative evidence of inefficiencies, which persist less due to technical necessity than to institutional path dependencies and policy priorities favoring supply expansion. Interpreting these outcomes through hydrosocial and water justice perspectives therefore offers deeper insight into the structural drivers of water transfers. Linking quantitative indices with qualitative evidence further demonstrates that measures of “efficiency” are embedded in, and shaped by, broader political, institutional, and ecological processes.

### Efficiency *versus* equity trade-offs revealed by the indices

Recalling the main aspects of water security (water availability, productivity and economic activity, hazards, human and environmental health, and governance), the qualitative document analysis of the impacts of the water transfers shows that beyond the question of efficiency, Istanbul’s increasing reliance on water transfers has impacts for the city and the exporting provinces. Here, the quantitative indices provide a critical empirical contribution by demonstrating that water availability in the exporting provinces has measurably declined as transfer volumes increase, corroborating qualitative accounts of flow reduction and heightened drought sensitivity. The integration of quantitative and qualitative findings is particularly valuable: the near zero or negative DIO and SRI values for the donor provinces empirically validate the governance and justice concerns identified in the literature. The SRI values for Düzce for the Melen system transfer remain close to zero but increase slightly in 2010 during a low transfer amount (5.89 Mm^−3^) due to a period of drought across the region, showing that larger transfers increase water stress for the donor province of Düzce, while smaller transfers increase SRI values for both the receiving and exporting regions. This is in line with Duan et al.’s (2022) results where the SRI values for the largest IBTs in California coincided with drought years. The most efficient transfers across the USA overall, in Illinois, Wyoming, and Nevada, had smaller transfer magnitudes (<10 Mm^−3^) and SRI values exceeding 1.0 (thousand people Mm^−3^). For the Istranca system transfer, while close to zero, both the DIO and SRI values are negative and become increasingly inefficient as the transfer amount increases, alongside the population and water demand of Tekirdağ and Kırklareli (Figs. 3 and 4). This quantitatively demonstrates an efficiency-equity trade-off, where water security gains in Istanbul are achieved at the expense of heightened stress in donor basins.

Though there are enough current and planned water resources for the populations of Tekirdağ, Kırklareli, and Düzce (Varty Bayar, 2023), as affirmed by the relatively neutral SRI values for these provinces (Table 3, Figs. 4 and 5), future water security in donor basins will likely become more precarious. Index-based results suggest that efficiency declines nonlinearly as abstraction increases, indicating heightened vulnerability under future demand and climate stress. The development of Phase IV of the Melen Project will increase abstraction up to 62% of available water from Düzce, amplifying vulnerability when combined with rising population pressures, agricultural and industrial demand, and increased reliance on surface waters from the Melen watershed. This is comparable to Duan et al. (2022) who found the least naturally efficient IBTs in their USA study (*e.g.*, the Delaware aqueduct to New York City and Loup River power canal in Nebraska for irrigation) removed as much as 29%–58% of the streamflow from the water exporting watersheds. As Türkiye is in a climate change hotspot, future projections have shown that climate change will reduce water security in the region and Istanbul may face water challenges as early as the 2030s (Daloğlu Çetinkaya et al., 2022). This reinforces climate risk as a central constraint undermining the long-term viability of supply-side strategies.

### Governance and institutional lock-in sustaining reliance on water transfers

**Figure 6:**
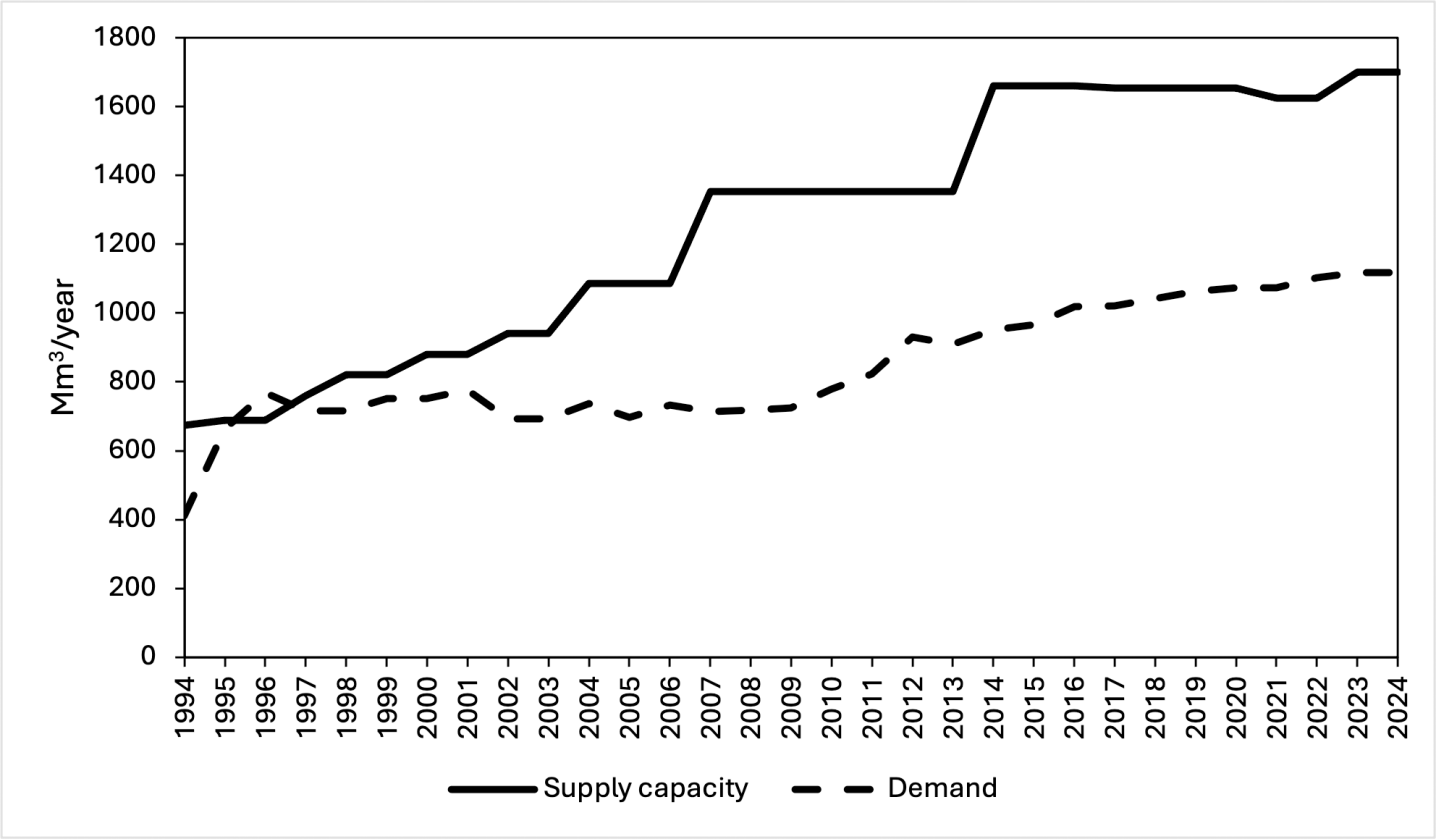
Temporal variations in Istanbul’s water supply capacity (including interbasin water transfers) and water demand (million m3/year). This depicts supply-demand dynamics where demand continues to grow despite increased supply capacity (ISKI Annual Reports 1994–2024). The straight line denotes supply capacity and dashed line denotes water demand.

The study also reveals how supply-side expansion reinforces a self-reinforcing supply–demand cycle. Increased water availability has enabled continued urban growth in Istanbul, contributing to land-use change, encroachment into forested and watershed areas, and emerging water-quality challenges, without curbing demand. For Istanbul, rapid population growth has driven the city’s water demand, particularly after increased in-migration in the 1980s and 1990s, while recent surges in tourism will also increase water use (İlhan, 2022). This dynamic is reflected in declining SRI values: as imported water volumes rise, marginal social benefits decrease, indicating diminishing returns of supply-side expansion. Assessing the reservoir effect and supply–demand cycles shows that despite the increase in Istanbul’s water supply and reservoir capacity, demand is steadily increasing as well, requiring more water resources (Fig. 6). Despite these risks, reducing reliance on water transfers is politically and infrastructurally challenging due to sunk costs, long asset lifetimes, inter-agency mandates, and the strategic framing of transfers as national-scale solutions. Nearly half of available water resource capacity for Istanbul comes from water transfers from outside of the city and in 2023, 58% of raw water came from the Melen and Istranca system transfers, 51% of this from the Melen system alone (ISKI, 2025; ISKI, 2023). This reliance was also embedded in the ISKI Master Plan for Drinking Water and Sewerage for Istanbul 2023-2053 which plans for the Melen system transfer to consistently provide 50% of Istanbul’s water demand by 2053 despite increased investments in other water resources and continued water demand management policies (ISKI’nin 2053 Planı Hazır , 2025; ISKI, 2023). This institutional lock-in explains why quantitatively inefficient transfers continue to be expanded despite declining index performance.

### Justice implications and hydrosocial dynamics

From a hydrosocial perspective, the findings align with studies showing how power relations are embedded in the physical and managerial dimensions of water governance (Islar & Boda, 2014). The historical evolution of Istanbul’s water-supply system follows patterns identified by Hommes & Boelens (2017), whereby IBTs reconfigure water ownership and regulation, institutionalize new territorial arrangements, and legitimize appropriation through discourses of scarcity. In this study, negative SRI values operationalize these dynamics by quantifying how water stress is redistributed from politically dominant urban centers to less powerful rural regions. This redistribution is not merely hydrological but institutional: Istanbul’s economic and political dominance has enabled metropolitan authorities, notably ISKI and the 14th Regional Directorate of DSI, to extend their influence beyond the city’s administrative boundaries to water-exporting provinces such as Kırklareli, Tekirdağ, Sakarya, and Düzce. Although environmental assessments acknowledge local water uses in donor basins, urban supply needs have consistently been prioritized in planning and implementation. The resulting justice implications span distributive, procedural, and recognition dimensions (IPCC, 2022). In distributive terms, donor regions bear ecological disruption, reduced flows, and heightened drought vulnerability, reflected in the consistently negative or near-zero SRI values for the Istranca and Melen system transfers. Procedurally, the expansion of metropolitan mandates weakens meaningful participation of local actors in decision-making processes affecting their water security. In terms of recognition, the historical and cultural relationships between rural communities and their waters are often overlooked. Together, these dimensions align closely with the negative social-efficiency outcomes quantified in this study, reinforcing the need for more equitable and inclusive governance frameworks for inter-basin water management.

Several limitations of the quantitative assessment should be acknowledged. First, the indices rely on annual data, it does not capture seasonal variability or short-term hydrological extremes. Where interpolation was required to address data gaps, this approach may smooth interannual variability and potentially dampen drought signals, particularly in drought-prone, semi-arid systems. Second, informal and industrial water uses could not be fully captured due to data constraints. Taken together, these uncertainties do not invalidate the results but indicate that the reported efficiency values should be interpreted with caution and can be considered conservative estimates of stress in donor basins. Additionally, this study differs from the original research conducted by Duan et al. (2022) for which they created these indices. Duan et al. (2022) compared IBTs from across the USA and developed a dynamic water stress model to understand regional water availability and stress levels under various contexts beyond the watersheds involved in the transfers. This allowed for an expanded analysis of the indices under different climate change scenarios, population and water demand changes, and streamflow variations. The availability of data to conduct these models allowed for Duan et al. (2022) to conduct a more comprehensive look at all transfers within the country and make recommendations for adaptation under future climate change scenarios. For the purposes of our paper and due to data limitations we only looked at the administrative boundaries for each transfer. Future studies could benefit from an expanded analysis upstream and downstream of the transfers in the Marmara region and the incorporation of future scenarios to understand these impacts to the environment and people across the broader region, particularly given the fact that Istanbul has relied on water transfers from additional sources in the past such as Sakarya.

Although path dependency of urban infrastructure is locked in with the reliance on current water transfers, there are still opportunities to manage water for environmental and socio-economic benefit for Istanbul and the donor provinces. ISKI, as well as the national and local governments, have demonstrated a greater interest in improving demand-side water management options such as through rainwater harvesting, encouraging reduced consumption, and reducing losses and leaks from the system, which would reduce dependence on the Istranca and Melen system transfers. Akalın, Mertoğlu & Ertürk (2025) recommend reducing per capita water use as an important governance tool, while Savun et al. (2021) found that reuse of greywater and continuing ISKI’s practice of irrigation with treated water are the most attractive alternatives. Lessons can be learned from historical water management where Roman, Byzantine, and Ottoman water supply systems utilized more sustainable water resources such as rainwater and managed population in-migration to reduce demand. ISKI also recognizes the need for a more organized and holistic water management, including the development of a Water Law and updated legislation to support Türkiye as a water stressed country (ISKI, 2023), which should include more involvement in decision making by those impacted by water supply infrastructure.

## Conclusions

This study employed a socio-hydrological approach to assess the water-security implications of Istanbul’s reliance on water transfers from outside the city’s boundaries, integrating quantitative water-security indices with qualitative analysis informed by hydrosocial and water-justice perspectives. While the indices provided insights into the relative efficiency and outcomes of the water transfers for both Istanbul and the donor basins (Tekirdağ, Kırklareli, Düzce), the qualitative evidence revealed environmental, socio- economic, and governance consequences that remain largely invisible in quantitative assessments alone. By triangulating these methods, the study offers a more holistic understanding of how these transfers shape regional water security across connected basins.

The findings highlight that Istanbul’s political and economic dominance reinforces a supply-side water management paradigm that prioritizes urban water security while externalizing costs to surrounding regions. This dynamic generates unequal burdens for donor basins and raises significant distributive, procedural, and recognition-justice concerns. By explicitly linking efficiency metrics with qualitative evidence of lived impacts, the study contributes a novel multi-scalar perspective on urban-rural water governance in Türkiye. Despite strong path dependencies associated with existing transfer infrastructure, recent policy shifts by ISKI and national authorities toward demand-side measures, including rainwater harvesting, leakage reduction, and efficiency improvements, signal emerging alternatives to continued supply expansion. Strengthening and scaling such approaches is critical to reduce long-term vulnerabilities, conserve ecosystems, and mitigate adverse impacts on local livelihoods. In this context, the establishment of cross basin governance mechanisms, such as permanent coordination councils involving Istanbul and water exporting provinces, could facilitate more transparent decision-making, shared monitoring, and joint planning across administrative boundaries.

In parallel, compensation and benefit-sharing mechanisms, such as targeted investments in water infrastructure, ecosystem restoration, and rural development in donor basins, could help address long-standing distributive inequities associated with the water transfers. Crucially, inclusive governance arrangements must ensure that donor-basin communities participate meaningfully in decision-making and benefit from development investments, rather than remaining passive suppliers of water to the metropolitan core.

Drawing on historical experience and emphasizing stakeholder-driven governance, the study advocates for institutional arrangements that integrate hydrological realities with social equity considerations, supporting integrated, climate-resilient, and justice-oriented water management that accounts for both urban and rural needs. Future research should extend this analysis by incorporating population dynamics and climate-change scenarios similar to the Duan et al. (2022) study, examining how demographic pressures and hydroclimatic extremes may reshape the sustainability, equity, and governance of inter-basin water transfers over time. Without such an integrated perspective, continued reliance on large-scale water transfer risks amplifying environmental degradation, social inequities, and systemic vulnerability across the wider region.

##  Supplemental Information

10.7717/peerj.21241/supp-1 Supplemental Information 1 Raw data for calculations

10.7717/peerj.21241/supp-2 Supplemental Information 2 Document analysis of research on Istanbul and the surrounding region’s water security and identified relevant main themes

## Additional Information and Declarations

### Data Availability

The following information was supplied regarding data availability: The raw data is available in the Supplementary File.

## References

[ref-1] Acara E (2019). Sequestering a river: the political ecology of the dead Ergene River and neoliberal urbanization in today’s Turkey. Annals of the American Association of Geographers.

[ref-2] Akalın N, Mertoğlu B, Ertürk A (2025). Assessing the future water potential of Istanbul and the need for inter-basin water transfer and the trade-offs for water allocation. Water Supply.

[ref-3] Aktaş O (2014). Impacts of climate change on water resources in Turkey. Environmental Engineering and Management Journal.

[ref-4] Bakırcı M (2016). The effects of dams on the spatial reorganization: the case of Melen Dam. Marmara Cografya Dergisi.

[ref-5] Bekiroğlu S, Eker O (2011). The importance of forests in sustainable supply of drinking water: Istanbul example. African Journal of Agricultural Research.

[ref-6] Bowen GA (2009). Document analysis as a qualitative research method. Qualitative Research Journal.

[ref-7] Burak S, Bilge A, Ülker D (2021). Assessment and simulation of water transfer for the megacity Istanbul. Physical Geography.

[ref-8] Carr G, Barendrecht MH, Balana BB, Debevec L (2022). Exploring water quality management with a socio-hydrological model: a case study from Burkina Faso. Hydrological Sciences Journal.

[ref-9] Caretta MA, Mukherji A, Arfanuzzaman M, Betts RA, Gelfan A, Hirabayashi Y, Lissner TK, Liu J, Lopez Gunn E, Morgan R, Mwanga S, Supratid S (2022). Water. In: Carr, G., Barendrecht, M.H., Balana, B.B., Debevec, L. 2022. Exploring water quality management with a socio-hydrological model: a case study from Burkina Faso. Hydrological Sciences Journal.

[ref-10] Cengiz S, Atmis E, Gormus S (2019). The impact of economic growth oriented development policies on landscape changes in Istanbul Province in Turkey. Land Use Policy.

[ref-11] Cinar T (2009). Privatisation of urban water and sewerage services in Turkey: some trends. Development in Practice.

[ref-12] Colby BG (1990). Transactions costs and efficiency in Western water allocation. American Journal of Agricultural Economics.

[ref-13] Conker A (2016). The power struggle in the layer of transnational hydropolitics: the case of the Ilisu Dam project. Eurasian Journal of Social Sciences.

[ref-14] Cook C, Bakker C (2012). Water security: debating an emerging paradigm. Global Environmental Change.

[ref-15] Daloğlu Çetinkaya I, Yazar M, Kilinc S, Guven B (2022). Urban climate resilience and water insecurity: future scenarios of water supply and demand in Istanbul. Urban Water Journal.

[ref-16] Devlet Su İşleri (DSI) (n.d). 11. Bölge Müdürlüğü. Toprak ve su kaynaklar1.

[ref-17] Devlet Su İşleri (DSI) (n.d). 5. Bölge Müdürlüğü. Toprak ve su kaynaklar1.

[ref-18] Di Baldassarre G, Sivapalan M, Rusca M, Cudennec C, Garcia M, Kreibich H, Konar M, Mondino E, Maọtrd J, Pande S, Sanderson MR, Tian F, Viglione A, Wei J, Wei Y, Yu DJ, Srinivasan V, Blöschl G (2019). Sociohydrology: scientific challenges in addressing the sustainable development goals. Water Resources Research.

[ref-19] Di Baldassarre G, Wanders N, AghaKouchak A, Kuil L, Rangecroft S, Veldkamp TIE, Garcia M, Van Oel PR, Breinl K, Van Loon AF (2018). Water shortages worsened by reservoir effects. Nature Sustainability.

[ref-20] Dinçkal N (2008). Reluctant modernization: the cultural dynamics of water supply in Istanbul, 1885–1950. Technology and Culture.

[ref-21] Doğruel F, Doğruel AS (2018). Two phases of deindustrialization policies in Istanbul. Journal of Research in Economics.

[ref-22] Duan K, Caldwell PV, Sun G, McNulty SG, Qin Y, Chen X, Liu N (2022). Climate change challenges efficiency of inter-basin water transfers in alleviating water stress. Environmental Research Letters.

[ref-23] Düzce Valiliği (2012-2023). ll çevre durum raporu. T.C. Çevre, Şehircilik ve İklim Değişikliği Bakanlığı. https://ced.csb.gov.tr/il-cevre-durum-raporlari-i-82671.

[ref-24] Faundez M, Alcayaga H, Walters J, Pizarro A, Soto-Alarez M (2022). Sustainability of water transfer projects: a systematic review. Science of the Total Environment.

[ref-25] Güneralp B, Tezer A, Albayrak I, Elmqvist T (2013). Local assessment of Istanbul: biodiversity and ecosystem services. Urbanization, biodiversity and ecosystem services: challenges and opportunities: a global assessment.

[ref-26] Gupta J, vander Zaag P (2008). Interbasin water transfers and integrated water resources management: where engineering, science and politics interlock. Physics and Chemistry of the Earth.

[ref-27] Güvemli Ö (2017). Şile’de 12 köy sular altında kalacak. Sözcü. https://www.sozcu.com.tr/2017/gundem/silede-12-koy-sular-altinda-kalacak-1700047/.

[ref-28] Haeffner M, Hellman D, Cantor A, Ajibade I, Oyanedel-Craver V, Kelly M, Schifman L, Weasel L (2021). Representation justice as a research agenda for socio-hydrology and water governance. Hydrological Sciences Journal.

[ref-29] Harris LM, Islar M, Atasoy Y (2013). Neoliberalism, nature, and changing modalities of environmental governance in contemporary Turkey. Global economic crisis and the politics of diversity: transregional variations, mixed responses, new tensions.

[ref-30] He C, Liu Z, Wu J, Pan X, Fang Z, Li J, Bryan BA (2021). Future global urban water scarcity and potential solutions. Nature Communications.

[ref-31] Hommes L, Boelens R (2017). Urbanizing rural waters: rural–urban water transfers and the reconfiguration of hydrosocial territories in Lima. Political Geography.

[ref-32] IBB (2018). Istanbul climate change action plan summary report.

[ref-33] IBB (2021). Istanbul climate change action plan. Istanbul Büyükşehir Belediye. https://cevre.iBB.Istanbul/wp-content/uploads/2022/06/Istanbul_climate_change_action_plan_v03.pdf.

[ref-34] İlhan A, Genc M (2022). İstanbul’un suyu, İstanbul’un geleceği. İstanbul Su Kulturu.

[ref-35] İlhan A (2025). Expanding water, deepening injustice: how Istanbul’s IBWT projects reshape urban and rural lives. YILLIK: Annual of Istanbul Studies.

[ref-36] IPCC (2022). Climate change 2022: impacts, adaptation and vulnerability. Contribution of working group II to the sixth assessment report of the lntergovernmental panel on climate change.

[ref-37] ISKI (1994-2005). Faaliyet Raporları 1994–2005.

[ref-38] ISKI (2006-2024). Annual reports, 2006–2024. ISKI.

[ref-39] ISKI (2025). 1stanbul’un Su Kaynakları, ISKI. https://iski.istanbul/kurumsal/hakkimizda/su-kaynaklari.

[ref-40] ISKI’nin 2053 Planı Hazır (2023). Su ve cevre teknolojileri dergisi, 182. https://www.suvecevre.com/edergi/19/182/4/index.html.

[ref-41] Islar M (2012). Struggles for recognition: privatisation of water use rights of Turkish rivers. Local Environment.

[ref-42] Islar M, Boda C (2014). Political ecology of inter-basin water transfers in Turkish water governance. Ecology and Society.

[ref-43] Istanbul Master Plan Consortium IMC (1999). Istanbul water supply, sewerage and drainage, sewage treatment and disposal master plan.

[ref-44] Karakaya N, Evrendilek F, Gonenc E (2014). Interbasin water transfer practices in Turkey. Ecosystem & Ecography.

[ref-45] Keys PW, Wang-Erlandsson L, Gordon LJ (2018). Megacity precipitationsheds reveal tele- connected water security challenges. PLOS ONE.

[ref-46] Kırklareli Valiliği (2012-2023). 1l çevre durum raporu. T.C. Çevre, Şehircilik ve İklim Değişikliği Bakanlığı. https://ced.csb.gov.tr/il-cevre-durum-raporlari-i-82671.

[ref-47] McDonald RI, Mansur AV, Ascensão F, Colbert M, Crossman K, Elmqvist T, Gonzalez A, Güneralp B, HaaseDagmar Hamann M, Hillel O, Huang K, Kahnt B, Maddox D, Pacheco A, Pereira HM, Seto KC, Simkin R, Walsh B, Werner AS, Ziter C (2020). Research gaps in knowledge of the impact of urban growth on biodiversity. Nature Sustainability.

[ref-48] McGrane SJ (2016). Impacts of urbanisation on hydrological and water quality dynamics, and urban water management: a review. Hydrological Sciences Journal.

[ref-49] Mostert E (2018). An alternative approach for socio-hydrology: case study research. Hydrology and Earth System Sciences.

[ref-50] Ozis U, Alkan A, Ozdemir Y, Harmancioglu NB, Altinbilek D (2020). Water works of ancient civilizations. Water resources of Turkey.

[ref-51] Ozturk I, Erturk A, Ekdal A, Gurel M, Cokgor E, Insel G, Pehlivanoglu-Mantas E, Ozabali A, Tanik A (2013). Integrated watershed management efforts: case study from Melen Watershed experiencing interbasin water transfer. Water Science & Technology Water Supply.

[ref-52] Peker E (2023). Enabling widespread use of rainwater harvesting (RWH) systems: challenges and needs in twenty-first-century Istanbul. European Planning Studies.

[ref-53] Rollason E, Sinha P, Bracken LJ (2021). Interbasin water transfer in a changing world: a new conceptual model. Progress in Physical Geography.

[ref-54] Saatci AM (2013). Solving water problems of a metropolis. Journal of Water Resource and Protection.

[ref-55] Savun B, Erbay B, Hekimoglu M, Burak S (2021). Evaluation of water supply alternatives for Istanbul using forecasting and multi-criteria decision making methods. Journal of Cleaner Production.

[ref-56] Sivapalan M, Savenije HHG, Bloschl G (2012). Socio-hydrology: a new science of people and water. Hydrological Processes.

[ref-57] Sivri N, Cilingirturk AM, Seker DZ, Imamoglu Z, Ucan ON (2017). Prediction of water consumption in Istanbul by means of statistical forecasting models and Geographical Information Systems (GIS). Fresenius Environmental Bulletin.

[ref-58] Sözen S, Yuzer E, Duba S, Gokcekus H (2021). Water management for Istanbul: collapse or survival. Environmental Earth Sciences.

[ref-59] Tekirdağ Valiliği (2012-2020). 1l cevrȩ durum raporu. T.C. Çevre, Şehircilik ve İklim Değişikliği Bakanlığı. https://ced.csb.gov.tr/il-cevre-durum-raporlari-i-82671.

[ref-60] TUIK (2023a). https://data.tuik.gov.tr/Bulten/Index?p=Su-ve-Atiksu-Istatistikleri-2022-49607.

[ref-61] TUIK (2023b). https://data.tuik.gov.tr/Bulten/Index?p=Nufus-Projeksiyonlari-2023-2100-53699.

[ref-62] United Nations (2023). The United Nations World Water Development report 2023: partnerships and cooperation for water.

[ref-63] Van Leeuwen K, Sjerps R (2016). Istanbul: the challenges of integrated water resources management in Europe’s megacity. Environment, Development and Sustainability.

[ref-64] Varty Bayar F (2023). The impact of inter-basin water transfers on the water security of Istanbul and surrounding watersheds (Thesis No. 821800). Master’s thesis.

[ref-65] Ward K, Smith SD, Crapper M, Charlesworth S, Booth CA, Adeyeye K (2020). Using the Byzantine water supply of Constantinople to examine modern concepts of sustainability. Sustainable water engineering.

[ref-66] Wesselink A, Kooy M, Warner J (2016). Socio-hydrology and hydrosocial analysis: Toward dialogues across disciplines. Wiley Interdisciplinary Reviews-Water.

[ref-67] WWF Turkey (2012). Mega dreams, empty hopes. Report on IBTs. (Çılgın ruyalar, boş umutlar. Havzalararasısu transferi).

[ref-68] Xia J, Dong Y, Zou L (2022). Developing socio-hydrology: research progress, opportunities and challenges. Journal of Geographical Sciences.

