# Supplementary material for: Efficiency and equity of water transfers to megacities: reconfiguring urban water security in Istanbul
Source: PeerJ. 2026 Jun 17;14:e21241. doi: 10.7717/peerj.21241 (PMC13282946; doi:10.7717/peerj.21241)
Supplement: Supplemental Information 2 [file peerj-14-21241-s002.docx]

Appendix B: Document analysis of research on Istanbul and the surrounding region’s water security and identified relevant main themes.

| **Research** | **Purpose, methodology, and/or research aim** | Main theme(s) explored | | | |
| --- | --- | --- | --- | --- | --- |
|  |  | Environ and socio- economic | Supply- demand cycle | Governance challenges | Limited attention to donor provinces |
| Government and NGO reports | | | | | |
| ISKI (1994-2024) *Annual reports.* | History of Istanbul’s water supply, current resources, current actions Istanbul is taking to conserve water. |  | ✔ | ✔ |  |
| ISKI (2023) *Drinking water and sewerage master plan 2023-2053.* | Short, medium, and long term planning on the provision of drinking water, wastewater treatment, and stormwater services in Istanbul until 2053. |  | ✔ |  |  |
| IBB (2021) *Climate action plans.* | Background on current climate change risks and future plans and policies. |  | ✔ |  |  |
| IBB (2020) *Canal Istanbul workshop.* | Report from workshops with experts on the impacts of the proposed Canal Istanbul project. | ✔ | ✔ |  |  |
| Istanbul, Kırklareli, Tekirdağ, and Düzce Valiliği (2012-2023) *Provincial environmental status reports.* | Information on water availability, water consumption, water quality, land use change etc. | ✔ | ✔ |  |  |
| IMC (1999) *Istanbul water supply, sewerage and drainage, sewage treatment and disposal master plan.* | Information on Istanbul’s water supply, environmental assessment reports etc. | ✔ | ✔ |  |  |
| WWF (2012) *Mega dreams, empty hopes. Report on IBTs.* | Information on the impacts of IBTs across Turkey. | ✔ |  |  | ✔ |
| Academic research | | | | | |
| Acara (2019) *Sequestering a river: The political ecology of the “dead” Ergene River and neoliberal urbanization in today’s Turkey.* | Political ecology study on the transformation of Turkey’s water sector since 2000, particularly in the Thrace region. | ✔ |  | ✔ | ✔ |
| Akalın et al. (2025) *Assessing the future water potential of Istanbul and the need for inter-basin water transfer and the trade-offs for water allocation.* | WEAP analysis to study future water potential and demand of Istanbul and calculate the trade-offs between sectoral water use. |  | ✔ |  |  |
| Aktas (2014) *Impacts of climate change on water resources in Turkey.* | Desktop study on the impacts of climate change and water use on water resource availability. | ✔ | ✔ |  |  |
| Bakirci (2016) *The effects of dams on the spatial reorganization: The case of Melen Dam.* | Assessment of the impacts of the Melen dam to both geographical and social characteristics. | ✔ |  |  | ✔ |
| Bekiroğlu and Eker (2010) *The importance of forests in sustainable supply of drinking water: Istanbul example.* | Qualitative analysis to study Istanbul’s forestry management and its impact to water resources. |  | ✔ |  |  |
| Burak et al. (2021) *Assessment and simulation of water transfer for the megacity Istanbul.* | Network analysis to model Istanbul’s water supply and demand (1995-2100). |  | ✔ | ✔ |  |
| Cengiz et al. (2019) *The impact of economic growth oriented development policies on landscape changes in Istanbul Province.* | Study to assess the impact of urban policies affect green areas in Istanbul, using satellite images and DEM data. |  | ✔ | ✔ |  |
| Cinar (2009) *Privatisation of urban water and sewerage services.* | History of water and sewerage management in Turkey. |  |  | ✔ |  |
| Daloğlu Çetinkaya et al. (2022) *Urban climate resilience and water insecurity: Future scenarios of water supply and demand in Istanbul.* | WEAP analysis to study Istanbul’s water security under climate change and socio-economic scenarios. |  | ✔ |  |  |
| Güneralp et al. (2013) *Local assessment of Istanbul: Biodiversity and ecosystem services.* | Assessment of the main challenges Istanbul faces in biodiversity conservation and ecosystem services. | ✔ | ✔ | ✔ |  |
| Harris and Işlar (2013) *Neoliberalism, Nature, and Changing Modalities of Environmental Governance in Contemporary Turkey.* | Analysis of changing water governance policies in Turkey from the Ottoman era to the present. |  |  | ✔ |  |
| Ilhan (2022) *Istanbul’un suyu, Istanbul’un gelecegi.* | Background of Istanbul’s water security and challenges. | ✔ | ✔ | ✔ | ✔ |
| Ilhan (2025) *Expanding water, deepening injustice: How Istanbul’s IBWT projects reshape urban and rural lives.* | Evaluation of the impact of water transfers to Istanbul from an environmental justice perspective. | ✔ | ✔ | ✔ | ✔ |
| Işlar and Boda (2014) *Political ecology of inter-basin water transfers in Turkish water governance.* | Political ecology and hydrosocial approach to study IBTs to Istanbul and Ankara. | ✔ | ✔ | ✔ | ✔ |
| Karakaya et al. (2014) *Interbasin water transfer practices in Turkey.* | IBT environmental and socio-economic impacts study. | ✔ | ✔ | ✔ | ✔ |
| Ozturk et al. (2013) *Integrated watershed management efforts: Case study from Melen Watershed experiencing interbasin water transfer.* | A summary of the Melen Watershed Protection Action Plan. | ✔ | ✔ |  |  |
| Peker (2023) *Enabling widespread use of rainwater harvesting (RWH) systems: Challenges and needs in twenty-first- century Istanbul.* | An analysis of the challenges of widespread implementation of rainwater harvesting systems in Istanbul using a participator inquiry with water management actors. |  |  | ✔ |  |
| Saatci (2013) *Solving water problems of a metropolis.* | Background of Istanbul’s water security and challenges. |  |  | ✔ |  |
| Savun-Hekimoğlu et al. (2021) *Evaluation of water supply alternatives for Istanbul using forecasting and multi-criteria decision making methods.* | Multi-criteria decision making (MCDM) analysis combined with demand forecasting and interviews with experts to assess demand-side water management options. |  |  | ✔ | ✔ |
| Sözen et al. (2021) *Water management for Istanbul: Collapse or survival.* | Remote sensing to analyze land use/cover change around Büyükçekmece and Ömerli watersheds. |  |  | ✔ | ✔ |
| Van Leeuwen and Sjerps (2016) *Istanbul: the challenges of integrated water resources management in Europa’s megacity.* | A IWRM assessment of Istanbul’s water resources and management. |  |  | ✔ | ✔ |
